# Exploring the potential of single-metals (Cu, Ni, Zn) decorated Al_12_N_12_ nanostructures as sensors for flutamide anticancer drug

**DOI:** 10.1016/j.heliyon.2023.e20682

**Published:** 2023-10-11

**Authors:** Emmanuel U. Ejiofor, Joyce E. Ishebe, Innocent Benjamin, Gideon A. Okon, Terkumbur E. Gber, Hitler Louis

**Affiliations:** a Computational and Bio-Simulation Research Group, University of Calabar, Calabar, Nigeria; b Department of Chemical Sciences, Clifford University, Owerrinta, Nigeria; c Department of Pure and Applied Chemistry, University of Calabar, Calabar, Nigeria; d Bingham University Karu, Abuja Nigeria, Nigeria; e Centre for Herbal Pharmacology and Environmental Sustainability, Chettinad Hospital and Research Institute, Chettinad Academy of Research and Education, Kelambakkam 603103, Tamil Nadu, India

**Keywords:** *Sensor*, *Aluminium nitride*, *Flutamide*, *Adsorption*, *DFT*

## Abstract

In recent years, scientists have been actively exploring and expanding biosensor technologies and materials to meet the growing societal demands in healthcare and other fields. This study aims to revolutionize biosensors by using density functional theory (DFT) at the cutting-edge B3LYP-GD3BJ/def2tzsvp level to investigate the sensing capabilities of (Cu, Ni, and Zn) doped on Aluminum nitride (Al_12_N_12_) nanostructures. Specifically, we focus on their potential to detect, analyze, and sense the drug flutamide (FLU) efficiently. Through advanced computational techniques, we explore molecular interactions to pave the way for highly effective and versatile biosensors. The adsorption energy values of −38.76 kcal/mol, −39.39 kcal/mol, and −39.37 kcal/mol for FLU@Cu–Al_12_N_12_, FLU@Ni–Al_12_N_12_, and FLU@Zn–Al_12_N_12_, respectively, indicate that FLU chemically adsorbs on the studied nanostructures. The reactivity and conductivity of the system follow a decreasing pattern: FLU@Cu–Al_12_N_12_ > FLU@Ni–Al_12_N_12_ > FLU@Zn–Al_12_N_12_, with a band gap of 0.267 eV, 2.197 eV, and 2.932 eV, respectively. These results suggest that FLU preferably adsorbs on the Al_12_N_12_@Cu surface. Natural bond orbital analysis reveals significant transitions in the studied system. Quantum theory of atom in molecule (QTAIM) and Non-covalent interaction (NCI) analysis confirm the nature and strength of interactions. Overall, our findings indicate that the doped surfaces show promise as electronic and biosensor materials for detection of FLU in real-world applications. We encourage experimental researchers to explore the use of (Cu, Ni, and Zn) doped on Aluminum nitride (Al_12_N_12_), particularly Al_12_N_12_@Cu, for biosensor applications.

## 1 Introduction

Prostate cancer is a complicated disease that leads to death of men in the world today. Its onset is predominant in men aged 40 and above [1], and the increased incidence of prostate cancer has led to remarkable changes in diagnosis and treatment over the past century. the increased prevalence of prostate cancer has prompted significant advancements in its diagnosis and treatment. The prostate, a walnut-sized gland situated behind the base of the penis in males [2,3]**,** plays a vital role in producing seminal fluid, which supports and transports sperm [4]**.** As men age, the prostate often enlarges, which can lead to the development of prostate cancer [5]. This type of cancer is quite common, affecting approximately one in every nine men, with around 60 % of men at risk of developing or living with prostate cancer today [6,7].

Prostate cancer originates from certain cells within the prostate gland that become cancerous (malignant), and it can potentially spread to various parts of the body, including the bones, lymph nodes, liver, brain, lungs, and other organs [8,9]**.** Moreover, this condition may impact the bladder, erectile nerves, and the sphincter responsible for controlling urination [10,11]. Notably, prostate cancer can be an aggressive disease that rapidly spreads to other parts of the body. Several risk factors have been associated with its onset in males, including age, family history, obesity, hypertension, lack of exercise, and persistently elevated testosterone levels [12]**.** In the early stages, prostate cancer may be asymptomatic, showing no noticeable symptoms. However, as the disease progresses, symptoms may emerge, such as the presence of blood in urine or pelvic pain [13]**.** There have been associations between certain infections, such as chlamydia, gonorrhea, or syphilis, and the development of prostate cancer [14]**.**

Flutamide is a non-steroidal also anti-androgen drug, which has been extensively utilized in prostate cancer treatment [15]**.** The drug function by blocking the effect of androgen which is a male hormone in order to stop or prevent the growth of cancer cells [16]**.** Although flutamide have been developed and has proven to be very useful in the treatment of prostate cancer, it has some side effects associated with the frequent intake of the drug such as serious liver problems, continuous loss of appetite, yellowing of skin or eyes, Upper stomach pain, itching, and dark urine [[17], [18], [19]]**.** This numerous side effect has prompted scientists in recent years to venture into developing nano sensor materials to effectively and efficiently carry this drug into the cells with minimal or no side effect. One of such nano sensor material is the Al_12_N_12_ nanocage. The Al_12_N_12_ nanocage have been utilized by various scientists for biosensor, gas sensor and drug delivery purposes. Sadegh Kaviani et al. carried out a DFT study on the adsorption of alprazolam drug using B_12_N_12_ and Al_12_N_12_ nanocages as potential bio sensor materials for carriage of the drug into bio systems. Employing the B3LYP/6-31G(d,p) level of theory [20]**,** the authors concluded that B_12_N_12_ forms more stable complex with the drug and also from the FMO analysis conducted in both water and gas phase the B_12_N_12_ nanocage was projected as a better bio sensor for the detection of Alprazolam (ALP). Also, elucidated the adsorption of amphetamine drug using pristine and doped Al_12_N_12_ and Al_12_P_12_ nano cages [21]. The studies were conducted using DFT/WB97XD/6-311G (d,p) in both gas and water phases, and they revealed that Al12P12 exhibited higher binding energy than Al12N12 in both phases Additionally, Mg and Ga doped AlN and AlP nano cages demonstrated increased sensitivity to the amphetamine drug, indicating their potential as biosensor materials for amphetamine detection and drug delivery into bio cells [21]. Sheikhi and co-workers utilized B_12_N_12_ and Al_12_N_12_ as nano sensors for the investigation and adsorption analysis of Zolinza drug on the nano cages. The DFT and time-dependent DFT study (TDDFT) calculations at the M06-2X/6-311+G(d, p) level of theory demonstrated B_12_N_12_ fullerene's suitability as a drug carrier for delivering the Zolinza drug [22]. Louis et al. employed DFT for modeling Ca_12_O_12_, Mg_12_O_12_, and Al_12_N_12_ nanostructured materials as sensors for phosgene (Cl_2_CO). They observed relatively low energy gap, chemical hardness, and electron potential values in the C_2_ complex, indicating its lower stability but higher reactivity and conductivity [23]. DFT study on the adsorption and detection of the amphetamine drug by pure and doped Al12N12 and Al12P12 nano-cages. They concluded that Mg and Ga doped ALN and ALP nano-cages could serve as promising biosensors for amphetamine drug detection [24]. Similarly, [25]. performed theoretical studies on various nanocages (Al_12_N_12_, Al_12_P_12_, B_12_N_12_, Be_12_O_12_, C_12_Si_12_, Mg_12_O_12_, and C24) to evaluate their potential for detecting and adsorbing Tabun molecule. Their DFT and TD-DFT study revealed Al_12_P_12_, Be_12_O_12_, B_12_N_12_, and C24 as promising sensors for Tabun detection. Niknam et al. conducted a DFT calculation alongside van der Waals (vdW) approximations using the PBE-GGA method with a double zeta polarization (DZP) basis set to understand the delivery and adsorption abilities of the flutamide drug on zinc oxide nano sheet (ZnONS). The studies indicated that ZnONS could potentially serve as drug delivery material for cancer treatment [26]**.** Furthermore, Maysam Talab and co-workers investigated the efficacy of B3O3 monolayers as carriers for delivering the flutamide drug. The interaction analysis between the drug and the adsorbent revealed energetically favorable adsorption of flutamide on the B3O3 monolayer in gaseous medium, with a significant presence of alternation in Egap of the preferred monolayer in the intended systems [27]**.**

Herein, this research work is aimed at revolutionizing the field of biosensor by utilizing the power of density functional theory (DFT) at the cutting-edge B3LYP-GD3BJ/def2tzsvp level of theory. Our focus lies on unraveling the remarkable sensing capabilities of (copper-Cu, nickel-Ni, and zinc-Zn metals) doped on Aluminum nitride (Al_12_N_12_) nanostructures, with a specific emphasis on their potential to efficiently detect and deliver flutamide (FLU) drug molecule. The electronic properties, HOMO – LUMO analysis and quantum descriptors was used to ascertain the reactivity and stability of the surfaces towards the sensing of flutamide, the geometric structural bond length was introduced to examine the feasibility of adsorption of flutamide on the studied nanomaterials. The NBO analysis is used herein, to comprehend the second order perturbation stability and electron transfer strength of the interacting systems, QTAIM analysis were employed to study the nature and strength of interaction between the studied systems, sensor mechanism as well as the adsorption energy analysis were performed to vividly extend our understanding on the adsorption behavior and mechanism of the studied adsorbate and adsorbent [28]**.**

## 2 Computational details

The calculations in this study were performed using Gauss View 6.0.16 and Gaussian 16 software [29,30] at the B3LYP functional and def2tzvp basis set along with D3BJ empirical dispersion. The D3BJ dispersion correction terms as used herein have been reported literature to account for the weak interactions that occur between the adsorbent and the adsorbates hence the choice of the dispersion used herein. The aluminium nitride nanostructures with flutamide molecules were optimized and their geometry parameters reduced at the ground state by placing a net charge of 0 (Q = 0 lel) with a singlet multiplicity (M = 2S_T_ + 1 = 1) for FLU@Cu–Al_12_N_12_, FLU@Ni–Al_12_N_12_, and FLU@Zn–Al_12_N_12_using the density functional theory (DFT) [31,32]. The quantum theory of atom in molecule QTAIM analysis was done by employing the Multiwfn 3.7 program software [33]. The natural bond orbitals (NBO) computations were performed to evaluate the second order perturbation energy and charge transfer from the donor and acceptor orbitals via NBO 7.0 module [34] available in Gaussian 16. Chemcraft software was utilized for the visualization of the HOMO – LUMO iso-surface of the studied system [35]. To obtained information on the reactivity and stability as well as the sensitivity and conductivity of the studied system, frontier molecular orbital analysis were performed using the log files generated from the gaussian calculations. The highest occupied molecular orbital (HOMO) and lowest unoccupied molecular orbital (LUMO) were utilized for the calculation of the energy gap and the respective quantum descriptors. The quantum chemical descriptors including; Ionization Potential (IP), Electron Affinity (EA), chemical hardness (***η***), global softness (σ), chemical potential (***μ***) electrophilicity index (***ω***) and electronegativity (***χ***). Applying the Koopman's theorem [36], (1), (2), (4), (5), (6), (7), (8) was used for the calculation of the following electronic descriptors.

(1) $$\text{IP}={-E}_{\text{HOMO}}$$

(2) $$EA={-E}_{\text{LUMO}}$$

(3) Energygap=IP–EA

(4) $$\eta =\frac{E_{HOMO}-E_{LUMO}}{2}$$

(5) $$\sigma =\frac{1}{2\eta }=\frac{1}{E_{HOMO}-E_{LUMO}}$$

(6) $$\mu =\frac{E_{HOMO}+E_{LUMO}}{2}$$

(7) $$\omega =\frac{\mu^{2}}{2\eta }$$

(8) $$\left(\chi \right)=-\frac{E_{HOMO}+E_{LUMO}}{2}$$

The electronic properties of the studied molecules, including the highest occupied molecular orbital (HOMO), lowest unoccupied molecular orbital (LUMO), and energy gap (Egap) in electron volts (eV), were computed to assess the stability and reactivity of the molecules [37]**.** The conductivity and reactivity of FLU@Cu–Al_12_N_12_, FLU@Ni–Al_12_N_12_, and FLU@Zn–Al_12_N_12_ were analyzed based on their energy gap, which represents the difference between HOMO and LUMO energies. Previous studies have shown that a smaller energy gap indicates higher reactivity, while a larger energy gap indicates greater stability of the system [38]. According to the frontier molecular orbital theory, the HOMO acts as an electron donor, and the LUMO acts as an electron acceptor, values of the energies of the HOMO, LUMO, energy gap and the quantum chemical descriptors are presented in Table 2.

**Table 1:** Optimized parameters (Bond length) before and after adsorption of Flu on the modeled nanostructures with adsorption energy of the studied systems investigated at B3LYP-GD3BJ/def2tzvp level of theory.

systems	Bond label	Bond length (Å)		E_ads_ (Kcal/mol)	Solvation.
		Before ads.	After ads.		
Al_12_N_12_@Cu-Flu	N_22_ – Cu_55_	1.970	1.877	−38.757	−0.075
	Cu_55_ – O_40_	–	1.931		
	Al_7_ – O_41_	–	1.839		
Al_12_N_12_@Ni-Flu	Ni_55_ – O_39_	–	1.824	−39.388	−0.067
	Ni_55_ – N_22_	1.794	1.834		
	Ni_55_ – Al_11_	2.306	2.309		
Al_12_N_12_@Zn-Flu	Zn_55_ – O_39_	–	1.853	−39.368	−0.112
	N_22_ – Zn_55_	1.536	1.891		
	Al_17_ – O_40_	–	1.979

**Table 1(b):** Comparative Analysis of Published Articles' Findings on Interaction Energy, Band Gap, and Other Parameters. The table provides a comprehensive comparison of previously reported published articles, focusing on their findings related to interaction energy, band gap, and various other parameters.

S/No.	Title	Findings	Ref.
1	Transition Metal-Decorated B12N12–X (X = Au, Cu, Ni, Os, Pt, and Zn) Nanoclusters as Biosensors for Carboplatin	Chemisorption which correlates with strong exothermic reactions was observed for B_12_N_12__Os@carbo, B_12_N_12__Cu@carbo, and B_12_N_12__Ni@carbo with chemical adsorption energy values of −87.22, −40.16 and −31.38 kcal/mol as well as a boron nitride cage decorated with Zn metal with an adsorption energy value of −27.61 kcal/mol.	[48]
2	Comment on “Kinetics and Mechanistic Model for Hydrogen Spillover on Bridged Metal-Organic Frameworks”	Their results show that adsorption occurs on the aromatic carbon atoms with electronic binding energies *D*_e_ of 25–35 kcal/mol for both functionals and MOFs.	[49]
3	Computational Study of Molecular Hydrogen Adsorption over Small (MO2) n Nanoclusters (M = Ti, Zr, Hf; n = 1 to 4)	Chemisorption leading to formation of metal hydride/hydroxides is exothermic by −10 to −50 kcal/mol for the singlet, and exothermic by up to −60 kcal/mol for the triplet. The predicted energy barriers are less than 20 kcal/mol. Formation of metal dihydroxides from the metal hydride/hydroxides is generally endothermic for the monomer and dimer and is exothermic for the trimer and tetramer.	[50]
4	Anchoring the late first row transition metals with B_12_P_12_ nanocage to act as single atom catalysts toward oxygen evolution reaction (OER)	Frontier molecular orbitals (FMOs) analysis indicates that the designed catalysts have semi-conducting capabilities which facilitate the transfer of electrons. The calculated FMOs energy gap (H-L E_gap_) values range from 2.01 to 2.88 eV.	[51]
5	First row transition metal doped B_12_P_12_ and Al_12_P_12_ nanocages as excellent single atom catalysts for the hydrogen evolution reaction	Results show that all transition metals are chemisorbed on the support, with interaction energies ranging from −0.65 to −3.85 eV. The chemical adsorption as observed herein is in agreement with our phenomenal of adsorption	[52]

**Table 2:** presents the Ionization Potential (IP, eV), Electron Affinity (EA, eV), Chemical Potential (μ, eV), Global Hardness (η, eV), Global Softness (S, eV−1), and Electrophilic Index (ω, eV) for all systems calculated using the B3LYP-GD3BJ/def2tzvp level of theory.

COMPLEXES	E_HOMO_	E_LUMO_	Band gap(eV)	IP (eV)	EA (eV)	σ(eV)	η(eV)	μ(eV)	ω(eV)	***χ***(eV)
Al_12_N_12_@Cu	−4.471	−2.368	2.103	4.471	2.368	0.475	1.051	−3.411	5.551	3.411
Al_12_N_12_@Ni	−5.374	−2.381	2.992	5.374	2.382	0.334	1.496	−3.878	5.026	3.878
Al_12_N_12_@Zn	−6.364	−2.419	3.946	6.364	2.419	0.253	1.973	−4.391	4.888	4.391
FLU@Cu–Al_12_N_12_	−3.823	−3.557	0.267	3.823	3.556	3.742	0.134	−3.681	50.951	3.681
FLU@Ni–Al_12_N_12_	−5.339	−3.142	2.197	5.339	3.142	0.456	1.098	−4.240	8.186	4.240
FLU@Zn–Al_12_N_12_	−5.661	−2.729	2.932	5.661	2.729	0.341	1.467	−4.196	6.002	4.196

**Table 3:** Natural bond orbital (NBO) Donor – Acceptor analysis and second order highest stabilization energy of studied metal doped Aluminum nitrate (Al_12_N_12_) using DFT/B3LYP-GD3BJ/def2tzvp level of theory.

STRUCTURE	DONOR(i)	Acceptor(j)	E^(2)^kcal/mol	E(_2_)-E(i) a.u	F (c,j)a.u
Al_12_N_12_@CU	σ*Al_10_–N-19	σ*Al_10_–N23	35.74	0.02	0.115
	σ*Al_10_–N22	σ*Al_10_–N19	33.27	0.04	0.114
	σ*Al_12_–N20	σ*Al_12_–N17	28.43	0.02	0.114
Al_12_N_12_@Ni	σ Al_7_–N24	π**l_7_-N22	34.94	0.90	0.161
	σ*Al_10_–N19	σ*Al_10_–N23	31.61	0.08	0.134
	σ (1)Al_7_–N24	σ*Al_7_–N23	26.83	0.74	0.129
Al_12_N_12_@Zn	σ*Al_11_–N22	σ*Al_11_–N20	51.67	0.03	0.910
	σ*Al_11_–N22	σ*Al_11_–N21	38.73	0.03	0.085
	σ*(1)Al_7_–N24	σ*Al_7_–N23	32.01	0.70	0.132
FLU@Cu–Al_12_N_12_	σ*Al_11_–N22	σ*Al_11_–N21	55.81	0.03	0.106
	σ*Al_10_–N23	σ*Al_10_–N23	52.15	0.01	0.068
	σ*Al_10_–N19	σ*Al_10_–N23	31.04	0.08	0.130
FLU@Ni–Al_12_N_12_	σ l_10-_N22	σ*N22 Ni55	675.55	0.41	0.561
	σ Al_11_–N22	σ*N22–Ni55	454.29	0.42	0.473
	σ (1)Al_11_–N22	σ Al_7_–N22	348.20	0.40	0.461
FLU@Zn–Al_12_N_12_	σ *Al_10_–N23	σ *Al_7_–N23	96.31	0.01	0.086
	σ *Al_1_–N23	σ *Al_1_–N13	79.91	0.02	0.118
	σ *Al_1_–N23	σ *Al_1_–N15	59.55	0.02	0.109

To investigate the electronic mechanism between Flutamide and the Al_12_N_12_ nanocage following adsorption, the density of state (DOS) plots was thoroughly examined (see Table 3). The DOS plots for FLU@Cu–Al_12_N_12_, FLU@Ni–Al_12_N_12_, and FLU@Zn–Al_12_N_12_ systems were visualized using the Origin software [39,40]**.** To characterize the bond types, present in the interactions between the metal-doped cage and Flutamide, we conducted Quantum Theory of Atoms in Molecules (QTAIM) analysis and Non-Covalent Interaction (NCI) using the Multiwfn programme [41,42]. Topological parameters like density of all electrons (ρ) Laplacian of electron density (∇^2^ρ), Hamiltonian kinetic energy (K), Lagrangian kinetic energy (G), and potential energy density (V) as proposed by Bader was analyzed in this study to investigate the nature and strength of interaction between the studied adsorbate and the surfaces [43]**.** To gain a comprehensive understanding of the adsorption strength of FLU on the investigated nanostructured materials, we employed the Boys-Bernardi counterpoise method, a well-established approach designed to correct for the basis set superposition error (BSSE) in intermolecular interaction calculations. In conjunction with equation (9), and the corresponding results are provided in Table 1 [44]**.**

(9) $$E_{\text{ads}}=E_{\text{complex}}-\left(E_{\text{surface}}+E_{\text{Flutamide}}\right)$$

in order to fully comprehend the sensor behavior and gain the knowledge on the adsorption efficacy of aluminium nitride (Al_12_N_12_) nanocage towards efficiently sense flutamide mechanism were calculated herein in this study [45]**.**

## 3 Results and discussion

### 3.1 Structural analysis, adsorption studies and solvation energy

Fig. 1(a) and (b) depict the geometric structures of flutamide before and after its adsorption on metal-doped nanostructures MAl_12_N_12_ (M = Zn, Ni, and Cu). The analysis of these structures provides valuable insights into the stability, conformational changes, and lowest potential energy of the investigated system. Throughout this study, all optimizations were conducted using the B3LYP/gd3bj/def2tzvp level of theory, resulting in stable structures with the lowest potential energy, as previously reported [46]**.** Table 1 presents the structural parameters, specifically bond lengths, before and after the interactions between flutamide and the modeled nanostructures. The adsorption energies of the studied systems and the solvation energy which was obtained considering the solvents effects. The bond lengths, measured in Ångstroms (Å), indicate that the O–Cu and O–Al bond labels exhibit bond lengths of 1.931 Å and 1.839 Å, respectively, whereas the Ni–O bond label has a bond length of 1.824 Å. Similarly, the bond lengths for the O–Zn and O–Al bond labels are 1.853 Å and 1.979 Å, respectively. These findings align with the observations made by Mehboob et al. [47] and are consistent with the results reported in previously published articles that investigated similar adsorption energies. studying adsorption energy enables one to comprehend adsorption processes, design materials with desired adsorption properties, and explore surface phenomena, owing to this quest for the aforementioned characteristics, the adsorption energy of the studied systems were evaluated and an increase in the adsorption strength was observed as thus Al_12_N_12_@Cu-Flu < Al_12_N_12_@Zn-Flu < Al_12_N_12_@Ni-Flu with adsorption energies of −38.757 < −39.368 < −39.368 respectively providing fundamental insights into the interaction between molecules and surfaces. Investigating the Solvation energy is very crucial for understanding the interaction between the analyte and the sensor's recognition element, as well as understanding the solvents effects as it affects the sensitivity and selectivity of the biosensor. To that end the solvation energy of the studied systems was investigated and the results is similarly presented in Table 1. Additionally, a comparative analysis was conducted, considering the interaction energy band gaps of transition metal doped Al_12_N_12_ cages with the interaction energies/band gaps of already reported literature of similar cages like Al_12_P_12_ and B_12_P_12_. And the results are presented in Table 1b.

**Fig. 1(a):**
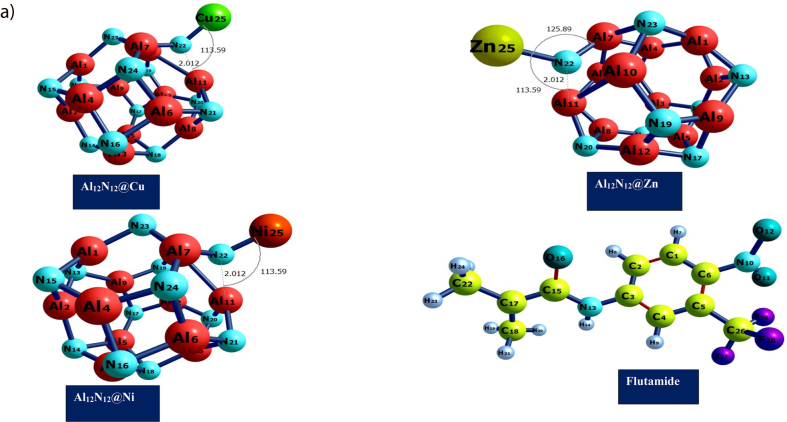
Optimized geometrical structures of the metal-doped surfaces Al_12_N_12_@Cu, Al_12_N_12_@Zn, Al_12_N_12_@Ni and the drug molecule Flutamide performed at B3LYP/GD3BJ/def2tzvp level of theory.

**Fig. 1(b):**
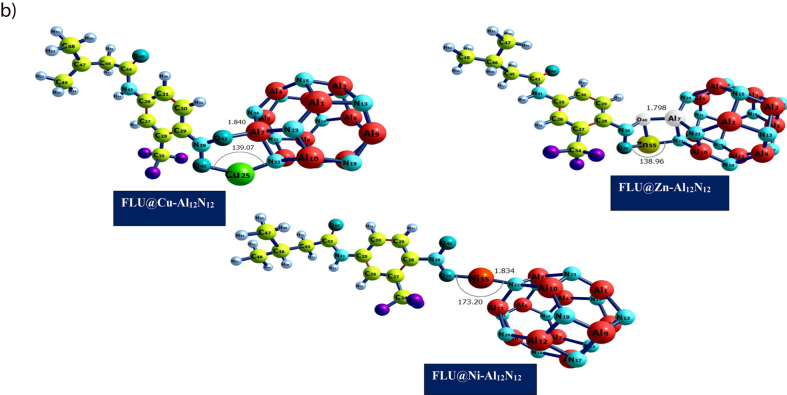
Optimized geometrical structures of flutamide (drug) and metal-doped surfaces FLU@Cu–Al_12_N_12_, FLU@Zn–Al_12_N_12_, and FLU@Ni–Al_12_N_12_ at the B3LYP/GD3BJ/def2tzvp level of theory.

The aluminum nitrite (Al_12_N_12_) consists of 12 aluminum and 12 nitrogen atoms which interacts to form a hexagonal and tetrahedron rings (Fig. 1) [48,49]. For all studied doped metals (Cu, Ni and Zn) with the Al_12_N_12_ after optimization, a bond distance of 1.970 Å existed between N–Cu, 1.794 Å existed between N–Ni, 2.306 Å existed between Al–Ni and 1.536 Å was seen between N–Zn. As seen in Fig. 1, the doped metals did not cause any structural change in the aluminum nitrite complex at the doping site. The increase in bond length for Al–Ni can be linked to larger atomic size of the metals [[50], [51], [52], [53]]. Another information revealed in Table 1 is the bond length after interaction of the doped surface with flutamide (FLU@Cu–Al_12_N_12_, FLU@Ni–Al_12_N_12_, and FLU@Zn–Al_12_N_12_), and pictorial representation of this interaction is shown in Fig. 2 below. The bond distance or length tell how stable the complex can exist. Generally, shorter bond distance suggests strong bond between the interacting atoms, making the complex stable and less susceptible to reactions with other chemicals or compounds according to Ref. [54]. After interaction, a longer bond distance was observed for the studied surfaces (Table 1). This can be linked to the effect of the drug flutamide on the doped metal surfaces. Oxygen was the only atom in flutamide that effectively formed bonding with the aluminum nitrite doped metal surface. Bond distance after interaction range from 1.824 to 2.309 Å. The highest bond length was seen Al_11_–Ni_55_, while the least bond length was seen at Ni_55_–O_39_.

**Fig. 2 (a):**
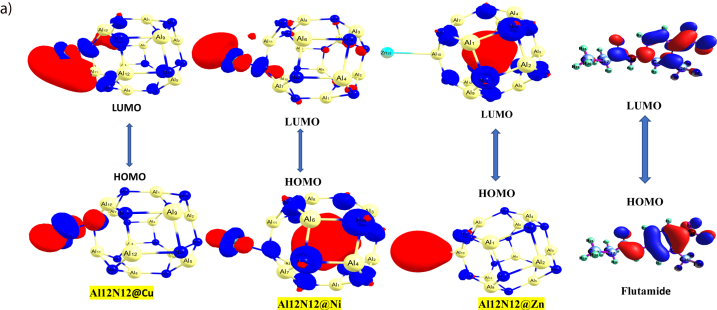
3Dimensional Maps of the HOMO/LUMO electrons distribution of the studied surfaces Al12N12@Cu, Al12N12@Ni, Al12N12@Zn and adsorbed drug molecule Flutamide obtained using Chemcraft software.

**Fig. 2 (b):**
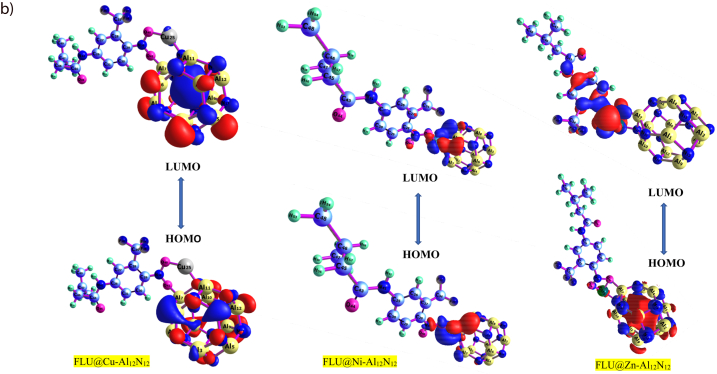
3Dimensional Maps of the HOMO/LUMO electrons distribution of the studied systems FLU@Cu–Al_12_N_12_, FLU@Ni–Al_12_N_12_

The key point in geometry analysis calculations is to establish the most stable configuration with minimum adsorption energy. To establish this, evaluation of all possible interactions is done by evaluating all bonds between the drug (flutamide) and the metal doped surfaces [55]. Flutamide, a cancer treatment agent consists of two electronegative atoms of fluorine and oxygen, suitable for nucleophilic attack. Result showed that the doped metals (Cu, Ni and Zn) were involved in bonding with oxygen atoms domiciled in flutamide. For the aluminum nitrite compound doped with Zn and Cu, oxygen was also seen forming bonds with the metals and aluminum. Studies have reported that electropositive metals after doping can initiate charge transfer, suggesting that the metals site can serve as points of interaction [56]**.** In this study, after optimization and interaction, flutamide successfully approached the metal (Al) atom of the aluminum nitrite complex, and also the doped metals. These results correlated with findings reported [57]. Table 1 also showed the adsorption energy (E_ads_) reported in Kcal/mole which was calculated computationally while the calculated and tabulated adsorption energy values of all studied complexes using the optimized structures obtained from the DFT/B3LYP/gd3bj/def2tzvp level of theory is reported on Table S1 of the supporting information. The adsorption energies were −38.757, −39.388, −39.368 kcal/mol for interactions of FLU@Cu–Al_12_N_12_, FLU@Ni–Al_12_N_12_and FLU@Zn–Al_12_N_12_ complexes respectively. Generally, a negative (-E_ads_) value suggests that the adsorption of flutamide onto the aluminum nitrite complexes with the doped metals is exothermic and energetically spontaneous [58]. The more negative the adsorption energy, the stronger the interaction occurs through the process of chemisorption's which connotes that strong adsorption potential as well as high sensitivity is present between the studied metal doped Al_12_N_12_ cage and FLU [59]. The influence of adsorption energy on bond distance is clearly documented in this study. Result showed that shorter bond distance after adsorption had the strongest interaction. The interaction Al_12_N_12_@Ni_Flu had a bond distance of 1.824 Å after adsorption and an E_ads_ of −39.388 kcal/mol. This suggests that as bond length reduces, energy of adsorption decreases. The results obtained here justify the aluminum nitrite doped metal complexes for the adsorption of flutamide drugs and as delivery agents.

### 3.2 Electronic properties

#### 3.2.1 HOMO-LUMO studies

**We further characterized the delivery material using the frontier molecular orbital (FMO) analysis. In the FMO analysis, the Highest Occupied Molecular Orbital (HOMO) and the Lowest Unoccupied Molecular Orbital (LUMO) are critical areas to be determined just as the iso-surfaces of the investigated systems are presented in**Fig. 2(a)**and**(b)**.** Furthermore, the difference between the HOMO and LUMO relative energies gives an idea of the ability of the delivery material and complex of studies [60]. The HOMO, LUMO, Band gap, E_FL_, and work function is presented in Table 2. The surface of the aluminum-nitrite complex when doped with Ni, Cu and Zn showed a band gap of 2.996 eV, 3.946 eV and 2.050 eV respectively. Flutamide the test drug showed a band gap of 4.506 eV. Interestingly, after interaction with the drugs, the band gap reduced giving a value of 0.267 eV, 2.196 eV, and 2.932 eV for FLU@Cu–Al_12_N_12_, FLU@Ni–Al_12_N_12_and FLU@Zn–Al_12_N_12_respectively. Similarly, the HUMO before interaction of the doped surfaces and flutamide was higher than the HOMO after interaction. For the LUMO, the reverse was the case at the studied functional. For Al_12_N_12_@Ni, the HUMO was −5.373 eV and LUMO was −2.381 eV. However, after interaction (FLU@Ni–Al_12_N_12_) the HOMO and LUMO were −5.338 eV and −3.142eV respectively. For Al_12_N_12_@Zn, the HOMO was −6.364eV and LUMO was −2.418 eV. After interaction (FLU@Zn–Al_12_N_12_), the HOMO and LUMO were −5.661 eV and −2.729 eV respectively. Similarly, Al_12_N_12_@Cu showed HOMO (−4.471 eV) and LUMO (−2.367 eV) before interaction. After interaction (FLU@Cu–Al_12_N_12_), HOMO and LUMO were −3.823 eV and −3.556 eV respectively. The interaction of the doped surfaces and the drug brought about a decrease in the HOMO and band gap, but led to an increase in LUMO. The reduction in energy after interaction suggests a higher conductivity and strong bonding of the drug on the doped surfaces. Studies have reported that large band gap relates to a low electrical conductivity and sensitivity of the system. However, it also indicates high stability of the system. The results obtained for band gap in the current study suggests that there is high sensitivity of the studied system, reflecting the delivery ability of the doped surfaces. The decrease in band gap after interaction further suggests that the metal doping enhanced the reactivity of the aluminum-nitrite complex and favored its electronic structure.

#### 3.2.2 Reactivity and stability descriptors

In this study, we conducted DFT calculations to explore the electronic properties of an absorbent molecule following drug absorption. Understanding the reactivity, conductivity, and stability of the materials is crucial for designing efficient drug sensors and drug delivery systems [61]. One key parameter we examined is the HOMO-LUMO energy gap, often referred to as the energy gap, which plays a vital role in determining the conducting potential of an electrochemical sensor material. An increase in conductivity results in a detectable electrochemical signal, confirming the absorption process and enabling drug detection [62]. Moreover, we evaluated the chemical global hardness value as an indicator of the structure's chemical stability and reduced chemical reactivity. Conversely, chemical softness demonstrated an inverse relationship with hardness, signifying increased chemical reactivity and reduced chemical stability of the structure. Additionally, we used the chemical potential to determine the stability of the investigated structure and the direction of electron transfer from the absorbate to absorbent. Furthermore, the electrophilicity index revealed an increase in chemical reactivity, while electronegativity helped determine the direction of electron flow in the chemical system, moving from a lower electronegative region to a higher one [63,64]. To calculate these properties, we applied the mathematical formulas detailed in equations (2), (4), (5) in the computational section. Koopmans's theorem was employed to obtain values for chemical hardness (***η***), global softness (σ), chemical potential (***μ***), and electrophilicity index (***ω***). The results of these calculations are recorded in Table 2.

According to the table, it is conspicuous that FLU@Cu–Al_12_N_12_ having the least energy gap records highest value of chemical softness of 3.742eV and lowest value of hardness calculated at 0.134eV while, FLU@Zn–Al_12_N_12_records the least global softness and high hardness values of 0.341eV and 1.467eV respectively. chemical potential (***μ***) was calculated as −3.681eV, −4.240eV and −4.196eV for FLU@Cu–Al_12_N_12_, FLU@Ni–Al_12_N_12_, and FLU@Zn–Al_12_N_12_ respectively. The reactivity of the studied system as observed from the energy gap followed an order FLU@Cu–Al_12_N_12_ > FLU@Ni–Al_12_N_12_ > FLU@Zn–Al_12_N_12_ > Al_12_N_12_@Cu > Al_12_N_12_@Ni > Al_12_N_12_@Zn with the respective energy gap values of 0.267 > 2.197 > 2.932 > 2.103 > 2.992 and 3.946 eV. Notably, the FLU@Cu–Al_12_N_12_ system exhibited the lowest energy gap, indicating its higher reactivity compared to the other systems. It is crucial to highlight that the incorporation of impurities such (Cu, Ni and Zn) into the Al_12_N_12_ nanostructure leads to a reduction in the energy gap, consequently enhancing the reactivity and conductivity of the examined systems. Likewise, the revised model has taken into consideration the influence of the solvent by implementing the Solvation Model Density (SMD) with water as the chosen solvent. The outcomes are documented in Table S2. Notably, the stability of the studied systems was found to be higher when optimized in water compared to the vacuum phase. This observation can be attributed to the solvent effect, likely resulting from the polar characteristics of water.

#### 3.2.3 Density of state (DOS)

**Fig. 3:**
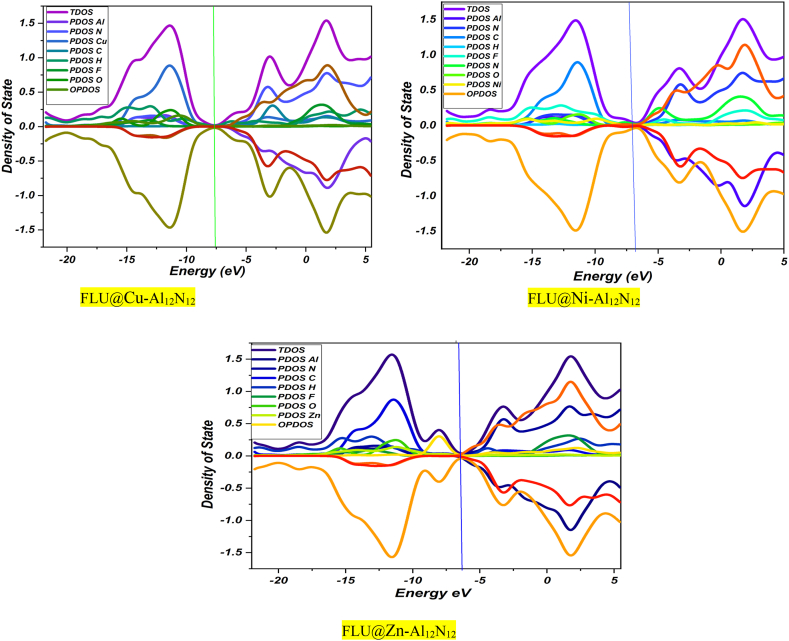
Density of state (DOS) plots for the different investigated systems FLU@Cu–Al_12_N_12_, FLU@Ni–Al_12_N_12_ and FLU@Zn–Al_12_N_12_ plotted using origin software.

To clearly defined the molecular orbital contribution as well as the participating atomic fragments within the metal doped aluminum nitrite cage (Al_12_N_12_) and the flu drug to effectively determine the sensing ability of the metal doped cages towards the delivering of the said drug. The analysis of interacting systems involved the use of three distinct density of state (DOS) plots. The first one, known as the Total Density of State (TDOS), was utilized to determine the energy gap. Additionally, the Partial Density of State (PDOS) was examined to identify contributions from molecular and fragment orbitals, which were observed on the left side of the graph. Moreover, the Overlap Density of State (OPDOS) was taken into account, typically observed on the right side of the plot [65,66]. The plots were visualized using the OriginLab 2018 software program to obtain a better graphics [67]. In the obtain plot the up spin observed in the positive region gives a mirror image of the different fragments present in this study which can be observed in the negative region of the plots. The fermi energy level observed was in close range with values −7.5, −6.5, 6.0 for Cu, Ni, and Zn doped Al_12_N_12_ interactions. The HOMO contribution in FLU@Cu–Al_12_N_12_shows that nitrogen has the major fragment contribution from −15.0 to −10.0 a.u and also dominant in the LUMO orbitals. Similarly, almost the same type of plot was observed for Al_12_N_12_@Ni_FLU and FLU@Zn–Al_12_N_12_respectively as seen in Fig. 3. Where carbon (C) atom fragment was seen to have the major orbital contribution both in the HOMO and LUMO orbitals.

#### 3.2.4 Natural bond orbital analysis (NBO)

In this research, we conducted a thorough investigation on the natural bonding orbitals between flutamide and Aluminum nitrate (Al_12_N_12_) using Density Functional Theory (DFT) computations at the B3LYP/def2tzvp level of theory. The analysis of natural bond orbitals is a valuable method that provides insightful understanding of intermolecular and intramolecular bonding, charge transfer stability, and the delocalization of π electrons from the donor to acceptor molecular orbitals [68]**.** This approach has proven to be an effective tool for interpreting charge transfer and hyper conjugative interactions within molecular systems [69]**.** The stability of the molecular system is attributed to the donor-acceptor interactions, where electron density is delocalized between occupied Lewis orbitals (bond or lone pair) and formally unoccupied non-Lewis orbitals (anti-bonding or Rydberg) [70]. In this study, we particularly focus on the second-order perturbation energy (E2) values derived from the Fork Matrix, which represent the stabilization energy of the donor-acceptor interactions. These values serve as a basis for understanding the strength of these interactions. A higher E2 value indicates a more intense and stronger donating ability from the donor to the acceptor, resulting in a significant range of conjugation throughout the entire system [71]**.** The computed stabilization energy associated with the delocalization of electrons between filled (i) and vacant (j) orbitals sheds light on the intermolecular and intramolecular hybridization and the confinement of electron density within the system [72]. In this work, all the results were extracted based on the highest E2 values, and the E2 values were mathematically determined according to equation (10), with the corresponding values recorded for further analysis.

(10) $${E\;}^{\left(2\right)}=\mathrm{qi}\frac{{\left(\mathrm{Fij}\right))}^{2}}{E\left(i\right)-E\left(j\right)}$$

From equation (10), qi denotes the electron donor orbital occupancy, Ei and Ej stands for the orbital energies of the donor and acceptor NBO orbital.

An interesting finding emerges from the analysis of the second-order perturbation energy in the Al_12_N_12_@Cu surface, where a notable major transition (σ→σ) is observed between σ Al10–N19 → σ* Al10–N23, σ* Al10–N22 → σ* Al10–N19, and σ* Al12–N20 → σ* Al12–N17, with corresponding energies of 35.74 kcal/mol, 33.27 kcal/mol, and 28.43 kcal/mol, respectively. This indicates significant stabilization through σ*→σ* transitions within the nanocage. Regarding the molecule flutamide, it exhibits three major transitions with energies of 34.94 kcal/mol, 31.61 kcal/mol, and 26.83 kcal/mol, respectively, occurring between σ Al7–N24 → π* Al7–N22, σ* Al10–N19 → σ* Al10–N23, and σ Al7–N24 → σ* Al7–N23. These interactions demonstrate the highest donating ability by σ*→σ* due to its corresponding highest E2 value. Upon adsorption at different points, distinct transitions are observed. The adsorption of the molecule on the nanocage (FLU@Cu–Al_12_N_12_) predominantly involves a major σ→σ* transition with values of 675.55 kcal/mol and 348.20 kcal/mol. A comparison of various adsorption points reveals that FLU@Ni–Al_12_N_12_ exhibits the highest energy, indicating intensive molecular interactions, electron delocalization, and a high level of conjugation. The stability of the adsorption points can be ranked as follows in terms of increasing stability: FLU@Ni–Al_12_N_12_ > FLU@Zn–Al_12_N_12_ > FLU@Cu–Al_12_N_12_.

### 3.3 Sensor mechanisms

Sensor mechanism is crucial to understanding the sensitivity, reactivity and adsorbing strength of the Al_12_N_12_ cage on sensing the FLU drug [73]**.** The performance of the studied cage in carrying the drug into bio cells depend largely on two parameters namely the energy gap (Δ*E*) and work function (Φ) [74]**.** According to Ref. [75]**.** For this study equation (11) was used to calculate the electrical conductivity in the studied system, this help in determining the sensitivity of the cage towards delivery of FLU. The equation clearly states that the electrical conductivity of any surface or absorbent which is combined with a chemical agent in the environment varies indirectly with the energy gap such that when the specie increases the energy gap tends to decrease [76]**.**

(11) $$\sigma =AT^{3/2}\;e^{\left(-\mathrm{Eg}/2\mathrm{kT}\right)}$$

from equation (11), σ is the electrical conductivity, A is the Richardson constant, T is the working temperature and k is the Boltzmann constant (2.0 × 10^−3^ kcal/mol). The influence of FLU on the fermi energy level and work function of the studied cage is investigated and reported on Table 4. According to Refs. [77,78], they defined the work function of a sensor material as the least amount of energy needed to move the weakest bond electron from the fermi energy level in a solid state to a vacuum space. The Φ gives us insights on the reactivity and sensitivity of Al_12_N_12_ in effectively sensing the studied FLU drug. The fermi energy level is described as the mid-point of the energy gap meanwhile, in the case of extrinsic semiconductors, the position of the Fermi level depends on the type of dopants and temperature [79]**.** It can be observed from the table that the HOMO, LUMO and Δ*E values of the metal doped Al12N12 cage changed substantially on the introduction of flutamide with* **%Δ*E*** value of −667.79 %, −36.43 % and −34.58 % for FLU@Cu–Al_12_N_12_, FLU@Ni–Al_12_N_12_and FLU@Zn–Al_12_N_12_respectively. Small values of energy gap are used to characterize materials with good conductivity. Promotions due to thermal excitation may occur and feasibility in transport of electron from the conduction band to the valence band. The work function is the inverse of the fermi energy level was calculated here using equation (12) [80]**.**

(12) $$\Phi =V_{el\;\left(+\infty \right)}\;–\;E_{\text{FL}}$$

**Table 4:** Theoretical calculation of electronic properties at DFT/B3LYP/def2tzvp level.

Surface	HOMO/eV	LUMO/eV	Band gap/eV	%Δ*E*	E_FL_/eV	WF (Φ)	%ΔΦ	Qt	ΔN	%ΔN
Flutamide	−7.296	−2.790	4.506	–	−1.395	1.395	–	–	–	–
Al_12_N_12_	−5.775	−3.404	2.370	–	−1.702	1.702	–	–	–	–
Al_12_N_12_@Ni	−5.373	−2.381	2.996	20.89	−1.189	1.189	−43.145	–	–	–
Al_12_N_12_@Zn	−6.364	−2.418	3.946	39.94	−1.200	1.200	−41.833	–	–	–
Al_12_N_12_@Cu	−4.471	−2.367	2.050	−15.61	−1.183	1.183	−43.872	–	–	–
FLU@Cu–Al_12_N_12_	−3.823	−3.556	0.267	−667.79	−1.778	1.778	33.465	−1.265	−0.147	−14.716
FLU@Ni–Al_12_N_12_	−5.338	−3.142	2.196	−36.43	−1.571	1.571	24.316	−0.807	−0.456	−45.587
FLU@Zn–Al_12_N_12_	−5.661	−2.729	2.932	−34.58	−1.364	1.364	12.023	−1.863	0.194	19.361

From equation (12), the V_el (+∞)_ is the vacuum electrostatic potential energy (which is assumed to be ≈ 0)**,** E_FL_ is the Fermi energy level**.** Taking that V_el (+∞)_ ≈ 0, therefore, Φ = –E_FL_. This entails that the work function is directly proportional to the negative value of the Fermi energy, which connotes that a change in the fermi energy will effect a change in the work function as can be seen in Table 4. According to Richardson Dushman. The variation in the work function of a semi-conductor can lead to a change in the field emission amount of a sensor material and was calculated in this study using equation (13) [81]**.**

(13) j=AT^(2)exp(−ΦkT)

where A = Richardson constant (A/m^2^), *k* = Boltzmann's constant, j = current density and T = temperature (K). equation (14) was used to calculated the change in work function after adsorption of the FLU drug [82]**.**

(14) $$\Delta \Phi =\left(\frac{\Phi_{2}-\Phi_{1}}{\Phi_{1}}\right)\;x\;100\%$$

where Φ_1_ and Φ_2_ are the values of the Φ for the doped Al_12_N_12_ and the flutamide drug/doped Al_12_N_12_ complex, respectively. Decrease in Φ was observed herein for the interactions involving the doped metals with FLU. The values as seen in Table 4 for the doped surfaces were 1.189eV, 1.200eV and 1.183eV for Cu, Ni and Zn doped surfaces respectively meanwhile on adsorption of the drug, the Φ observed was 1.778eV, 1.571eV and 1.364eV with observable changes of 33.47 %, 24.32 % and 12.02 % corresponding to FLU@Cu–Al_12_N_12_, FLU@Ni–Al_12_N_12_ and FLU@Zn–Al_12_N_12_. The changes observed in the energy gap and work function after adsorption in FLU@Cu–Al_12_N_12_, FLU@Ni–Al_12_N_12_ and FLU@Zn–Al_12_N_12_shows that they could be incorporated as a work - function type sensor materials for drug detection. To further estimate the electronic charge, and transfer strength present between the adsorbent and its adsorbate, equation (15) was used to calculated the charge transfer between the adsorbate and the adsorbent [83]**.**

(15) $$Q_{t}=Q_{\text{ads}}\;–\;Q_{\text{Isolated}}$$

in this context, Qads represents the charge of the adsorbate after adsorption, while Qisolated represents the charge of the decorated cage before any interaction. Additionally, for an electrochemical biosensor material, the biological binding and conductivity depend on event-dependent variations in resistance, capacitance, and conductance, along with temperature changes resulting from a thermally activated process. These factors play a crucial role in the design of biological sensor materials and were calculated in this study using equation (16) [84]**.**

(16) $$K=Ae^{–Ea/\;RT}$$

in equation (16), several important parameters are defined: K represents the rate constant, A is the pre-exponential factor, Ea denotes the activation energy, R stands for the universal gas constant, and T represents the absolute temperature. Furthermore, equation (17) is employed to determine the fraction of electron transfer, which serves as an indicator of the electronegativity and chemical hardness in both the surface and the adsorbed interaction [85]**.**

(17) $$\Delta N=\chi_{\text{sur}}\;–\;\chi_{\text{abs}}/2\left(\eta_{\text{sur}}\;–\;\eta_{\text{abs}}\right)$$

Furthermore, the negative adsorption energies observed in this study indicate the presence of exothermic interactions between the metal-doped surfaces and the drug. In accordance with transition metal theory, the strong adsorption strength of the adsorbate (FLU) on the adsorbent surface implies a challenging desorption process and an extended recovery time of the sensor material upon FLU adsorption. A longer recovery time is expected to be derived when there is increase in negative adsorption energies in terms of magnitude and was calculated in this study using equation (18) [86]**.**

(18) $$\tau =A^{-1}\;e\left(-\mathrm{Eads}/\mathrm{kT}\right)$$

Equation (18) can be explained as follows: In the equation, A, T, and k represent the attempt frequency, temperature, and Boltzmann's constant (∼2.0 × 10^-3 kcal/mol·K), respectively.

### 3.4 Topology analysis

#### 3.4.1 Bader quantum theory of atoms in molecules (QTAIM)

**Fig. 4:**
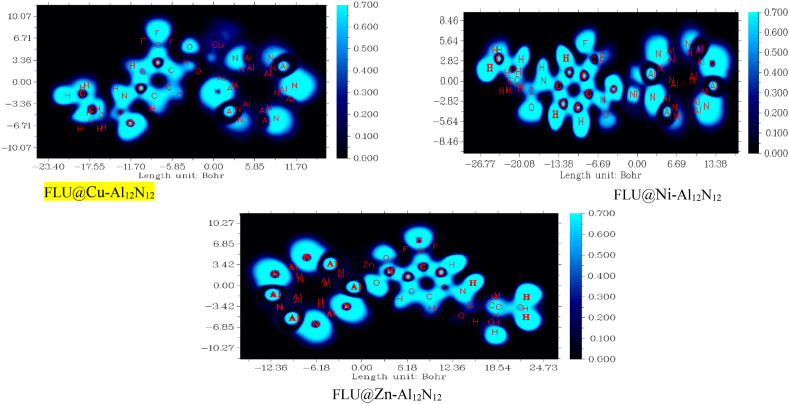
ELF plots of FLU@Cu–Al_12_N_12_, FLU@Ni–Al_12_N_12_, and FLU@Zn–Al_12_N_12_ systems illustrating the distribution of the electron density.

We employed the topology analysis as described by Bader in this study to evaluate the nature of interaction and the bond formation between the doped monolayer of Al_12_N_12_Cu, Al_12_N_12_Zn, Al_12_N_12_Ni and its adsorbate flutamide using some globally accepted function of topological parameters; Lagrangian kinetic energy G(r), The investigation in this study involved analyzing several properties related to electron density and interaction energies in different bond critical points (BCPs) of the studied systems [87]. The properties examined include the electron density ρ(r), potential energy density H(r), Hamiltonian kinetic energy K(r), Laplacian of electron density ∇2ρ(r), Electron localization function (ELF), energy density H(r), wave function values for orbitals (Ψorbital), ellipticity of electron density (e), and eigenvalues of Hessian (λ1, λ2, and λ3). Table 5 presents the topology parameters calculated in this research, while Fig. S1 in the supporting information displays the QTAIM molecular graphs for all the complexes studied, along with the bond critical points (BCPs) interactions. This graphical representation provides insights into the types of interactions (bonds) formed between the adsorbent and adsorbate and offers a clear understanding of the delivery ability of the materials investigated. The electron density ρ(r) values indicate the strength of interactions, with higher values suggesting strong chemical bonding between the studied system [88]. The values of *ρ(r)* in this study ranged from 0.110 to 0.811 a.u which indicated strong electrostatic interactions. Generally, interactions where *ρ(r)* > 0.1 a.u is considered covalent whereas, *ρ(r)* < 0.1 a.u is termed a weak non-covalent interaction [89]**.** From the values obtained from this study for *ρ(r)* in this study suggests that the systems showed covalent interactions which favors the use of the complex as a possible delivery agent for the studied drug. The ∇^2^*ρ (r)* in this study, ranged from 0.226 to 0.732. following the QTAIM theory, when ∇^2^*ρ (r)* = 0, a bond critical point assumes on the bond path connecting two interacting atoms**.** Generally, the Laplacian electron density reflects the possibility of electron density to accumulate or deplete at a point through space with studied systems [90]. Furthermore, when ∇^2^*ρ (r)* < 0 is seen, strong shared shell interatomic interaction is displayed by localized concentrations of the electron density distribution. Alternatively, weak closed shell bonds display local depletion when ∇^2^*ρ(r)* > 0. Studies have reported that high number of electron density at the bond critical points suggests good stability in terms of structure for the delivery material [91]**.** Numerous data about the attraction and repulsion of the decorated surface and the drug (flutamide) is indicated as shown by the second Eigen value. Negative and positive values were seen, and previous studies indicates that positive values of λ_2_ is implicative of steric repulsion which is destabilizing interaction, while negative values suggest an attractive force which is stabilizing interaction [92]**.** In this study, the only positive value (0.992) was seen at Cu_25_–O_41_ interaction for Al_12_N_12_@Cu_FLU system. More negative values were observed which suggest attractive forces stabilizing the interaction, further confirming the ability of the complex to serve as a delivery material. Negative values of H(r) indicating covalent interactions were observed in all studied systems and bonds. Additionally, the local electron localization function (ELF) density, associated with significant kinetic energy density and correlated to Pauli repulsive force, provided insights into the distribution of electron density within the bonds. An ELF value > 0.5 signifies covalent interactions, while an ELF value < 0.5 indicates non-covalent (closed shell) interactions, as shown in Fig. 4. The bond ellipticity (ϵ) was measured to assess the anisotropy of electron density in the bonds, and all systems exhibited a ϵ value lower than 1, confirming the stability of the studied complexes [93,94]. The results obtained from QTAIM in this investigation further support the stability of the complexes and their delivery potential.

**Table 5:** Presents the calculated values for various electronic properties of the studied complexes using the B3LYP/def2tzvp level of theory. The table includes electron density ρ(r), Laplacian electron density ∇2(r), Lagrangian kinetic energy G(r), Potential electron energy density V(r), Total electron energy density H(r), Eigenvalues (λ1, λ2, λ3), and Ellipticity (ϵ), all given in atomic units (a.u.).

System	Bond	BCP	ρ (r)	∇^2^ρ (r)	G(r)	K(r)	V(r)	H(r)	ELF	λ_1_	λ_2_	λ_3_	*ϵ*
FLU@Ni–Al_12_N_12_	Ni_55_–O_39_	91	0.114	0.732	0.207	0.249	−0.232	−0.249	0.122	0.104	−0.158	−0.159	0.008
FLU@Cu–Al_12_N_12_	Cu_25_–O_40_	152	0.811	0.465	0.125	0.897	−0.134	−0.897	0.108	0.706	−0.146	−0.949	0.540
	Cu_25_–O_41_	136	0.261	0.698	0.204	0.296	−0.233	−0.296	0.954	−0.106	0.992	−0.188	0.773
FLU@Zn–Al_12_N_12_	Zn_55_–O_39_	149	0.110	0.635	0.182	0.238	−0.206	−0.238	0.138	0.957	−0.176	−0.162	0.081
	Zn_55_–O_40_	137	0.528	0.226	0.641	0.740	−0.715	−0.740	0.100	0.502	−0.565	−0.517	0.092

#### 3.4.2 NCI analysis

**Fig. 5:**
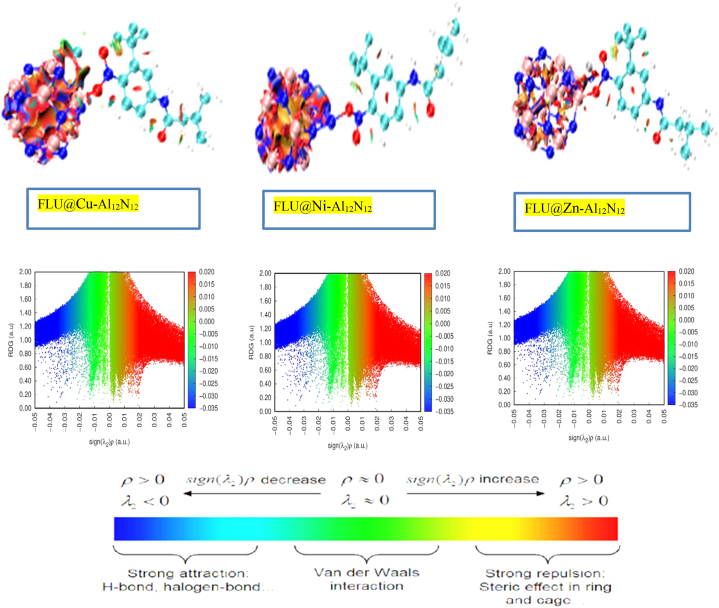
The NCI 2D and 3D isso-surface plots for all studied system FLU@Cu–Al_12_N_12_, FLU@Ni–Al_12_N_12_ and FLU@Zn–Al_12_N_12_ obtained using VMD.

The analysis utilized both 3D isosurface plots and 2D Reduced Density Gradient (RDG) scatter plots (Fig. 5) [90]. The 3D-RDG plots were valuable in understanding the 2D plots, particularly when the eigenvalue sign (***λ***) was less than zero (<0), indicating non-covalent interactions. The Non-covalent Interactions (NCI) analysis served as a crucial topological parameter to confirm the nature of intermolecular interactions between the metal-doped (Cu, Ni, and Zn) Al12N12 and the Flutamide drug in closed-shell systems. This analysis visualized all interactions contributing to the molecule's stabilization and allowed the differentiation between hydrogen bonds, weak van der Waals (vdW) forces, and repulsive steric or electrostatic interactions within the interacting system [95]. For this study, an iso-value range of 0.001–0.05 electron density units was used for NCI analysis. To interpret the results, the RDG graph was plotted against the product of the electron density (ρ) and the sign of the second eigenvalue of the Hessian matrix (***λ***). When ***λ*** > 0, the interaction was characterized as electrostatic, whereas ***λ***2ρ = 0 indicated an intermediate and relatively weak vdW interaction [96,97]. The green color between blue and red near the zero mark on the horizontal axis represented weak interactions with low electron density, indicative of weak van der Waals interactions due to non-directional and specific charge movements. On the other hand, the red isosurface zones indicated stronger interactions, primarily due to steric repulsion, although it could lead to the distortion of ion and molecule reactivity and conformation. Scattered maps observed in the negative regions of the eigenvalue (***λ***2), shown by the blue color on the horizontal axis of the isosurface graph, indicated strong attractive intermolecular interactions, including the presence of hydrogen bonds and interaction stability.

[[98], [99], [100]]**.** These colors are also discovered in the 2D – plot as seen below which helps explain the 3D isosurface better although they carry the same interpretation. In the plots as observed below, more of steric interactions represented by the red color was observed just in between the systems and was more in Al_12_N_12_@Cu_FLU, followed by FLU@Ni–Al_12_N_12_this explains the covalent interactions present in the interacting complexes. The 3D isosurface plot also correspondingly shows more of red colors as can be observed in the interactions.

## 4 Conclusions

Theoretical calculations were employed to study the interactions of flutamide drug with pure AlN nanostructure and its single metal doped surfaces Cu, Ni and Zn utilizing density functional theory at the B3LYP/GD3JB/def2tzsvp level of computational theory. The different analysis employed herein in this studies including the geometric structural, adsorption energy, frontier molecular orbital (FMO), global quantum descriptors, Electron localization function (ELF), density of states (DOS), natural bond orbital (NBO), quantum theory of atoms in molecules (QTAIM), non-covalent interactions (NCI), and sensor mechanism analysis were used to gain full knowledge on the interactions and adsorption possibilities of flutamide (FLU) on Cu, Ni and Zn doped Al_12_N_12_ nanocages. The results from adsorption of flutamide on FLU@Cu–Al_12_N_12_, FLU@Ni–Al_12_N_12_ and FLU@Zn–Al_12_N_12_showed respective energy values as thus; −38.76 kcal/mol, −39.39 kcal/mol, −39.37 kcal/mol respectively indicating the FLU chemically adsorbed on the studied nanostructures. From the FMO analysis we observed that FLU@Cu–Al_12_N_12_had the least energy gap of 0.267eV which connotes its high reactivity and less stability while FLU@Ni–Al_12_N_12_and FLU@Zn–Al_12_N_12_ was more stable on adsorbing Flutamide with a stronger electrophile. The electronic properties like the electron localization function (ELF) and density of state (DOS) showed the contributions of the metal doped nanostructures interactions with flutamide. Also evident from the HOMO - LUMO visualized plots, we noticed a major transfer of electron from the donor (HOMO) to acceptor (LUMO) molecular orbitals majorly in FLU@Ni–Al_12_N_12_ and FLU@Zn–Al_12_N_12_, and strong interactions was observed within the doped metals and the drug in the HOMO and strong interactions on the cage in the acceptor LUMO orbital. The sensor mechanism effectively showed that the sensing capacity of Cu, Ni and Zn doped AlN surfaces as promising biosensor sensor materials for flutamide and can be seen that as the energy gap reduces, work function increased and vice versa. Topological analysis such as the QTAIM and NCI reveals that covalent bonds were formed between interactions. Furthermore, the adsorption energy analysis calculated at the B3LYP/GD3BJ/def2-TZSVP level of theory showed that flutamide drug chemisorbed on the doped nanoclusters and could be considered as an effective, suitable and efficient sensing material for the transportation of flutamide into bio cells.

## Funding

This research was not funded by any Governmental or Non-governmental agency.

## Declaration of competing interest

The authors declare that they have no known competing financial interests or personal relationships that could have appeared to influence the work reported in this paper.
