# Supplementary material for: Exploring the potential of single-metals (Cu, Ni, Zn) decorated Al12N12 nanostructures as sensors for flutamide anticancer drug
Source: Heliyon. 2023 Oct 11;9(10):e20682. doi: 10.1016/j.heliyon.2023.e20682 (PMC10589786; doi:10.1016/j.heliyon.2023.e20682)
Supplement: Multimedia component 1 [file mmc1.docx]

**Supporting information**

**Exploring the potential of single-metals (Cu, Ni, Zn) decorated Al_12_N_12_ nanostructured as sensors for flutamide anticancer drug**

**Emmanuel U. Ejiofor ^a,b^, Joyce E. Ishebe^d^, Innocent Benjamin ^a^, Gideon A. Okon ^a, b^, Terkumbur E. Gber^a,b*^ and Hitler Louis ^a, c^***

*^a^* *Computational and Bio-Simulation Research Group, University of Calabar, Calabar, Nigeria*

*^b^Department of Chemical Sciences, Clifford University, Owerrinta*

*^c^ Department of Pure and Applied Chemistry, University of Calabar, Calabar, Nigeria*

*^d^ Bingham University Karu*

*^a^* *Computational and Bio-Simulation Research Group, University of Calabar, Calabar, Nigeria*

*^b^Department of Chemical Sciences, Clifford University, Owerrinta*

*^c^ Department of Pure and Applied Chemistry, Faculty of Physical Sciences, University of Calabar, Calabar, Nigeria*

*^d^ Department of Medical Laboratory Science, Knowledge University, Erbil, Iraq*

*^e^ Department of Chemistry, Tshwane University of Technology, Pretoria, South Africa*

**^**^Corresponding author’s email**: [louismuzong@gmail.com](mailto:louismuzong@gmail.com) and gberterkumburemmanuel@gmail.com

**TABLE S1**. Calculated Adsorption energy of the systems, calculated using the B3LYP/gd3bj/ def2tzvp level of theory.

| **Interaction** | **E_complex_** | **E_surface_** | **E_flutamide_** |
| --- | --- | --- | --- |
| Al_12_N_12_@Cu_FLU | -6308.25 | -5208.57 | -1060.92 |
| Al_12_N_12_@Ni_FLU | -6176.69 | -5076.38 | -1060.92 |
| Al_12_N_12_@Zn_FLU | -6447.79 | -5347.45 | -1060.92 |

**Table S2.** Ionization Potential (IP, eV), Electron Affinity (EA, eV), Chemical Potential (μ, eV), Global Hardness (η, eV), Global Softness (S, eV^−1^), and Electrophilic Index (ω, eV) calculated at for all systems estimated using the B3LYP-GD3BJ/def2tzvp level of theory in Water Phase.

| System | E_HOMO_ | E_LUMO_ | Band gap(eV) | IP (eV) | EA (eV) | σ(eV) | η(eV) | μ(eV) | ω(eV) | EFL |
| --- | --- | --- | --- | --- | --- | --- | --- | --- | --- | --- |
| Al_12_N_12_@Cu | -3.5658 | -1.9464 | 1.6194 | 3.5658 | 1.9464 | 0.6175 | 0.8097 | -2.7561 | 4.6908 | 2.7561 |
| Al_12_N_12_@Ni | -4.5658 | -2.9464 | 2.6194 | 4.5658 | 2.9464 | 0.4175 | 0.6097 | -3.7561 | 4.1256 | 3.7561 |
| Al_12_N_12_@Zn | -5.0055 | -2.0267 | 2.9788 | 5.0055 | 2.0267 | 0.3357 | 1.4894 | -3.5161 | 4.1503 | 3.5161 |
| FLU@Cu-Al_12_N_12_ | -5.0061 | -2.0607 | 2.9454 | 5.0061 | 2.0607 | 0.3395 | 1.4727 | -3.5334 | 4.2388 | 3.5334 |
| FLU@Ni-Al_12_N_12_ | -4.4287 | -3.1209 | 1.3078 | 4.4287 | 3.1209 | 0.7647 | 0.6539 | -3.7748 | 10.8955 | 3.7748 |
| FLU@Zn-Al_12_N_12_ | -4.3364 | -3.6934 | 0.6430 | 4.3364 | 3.6934 | 1.5552 | 0.3215 | -4.0149 | 25.0690 | 4.0149 |


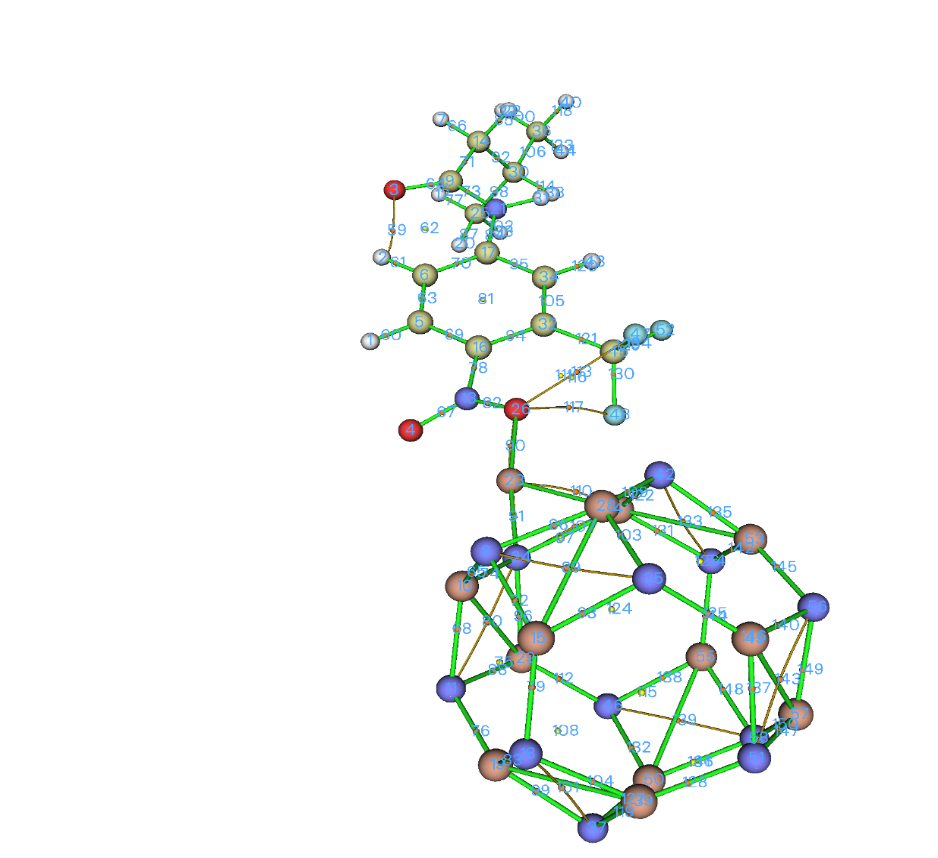

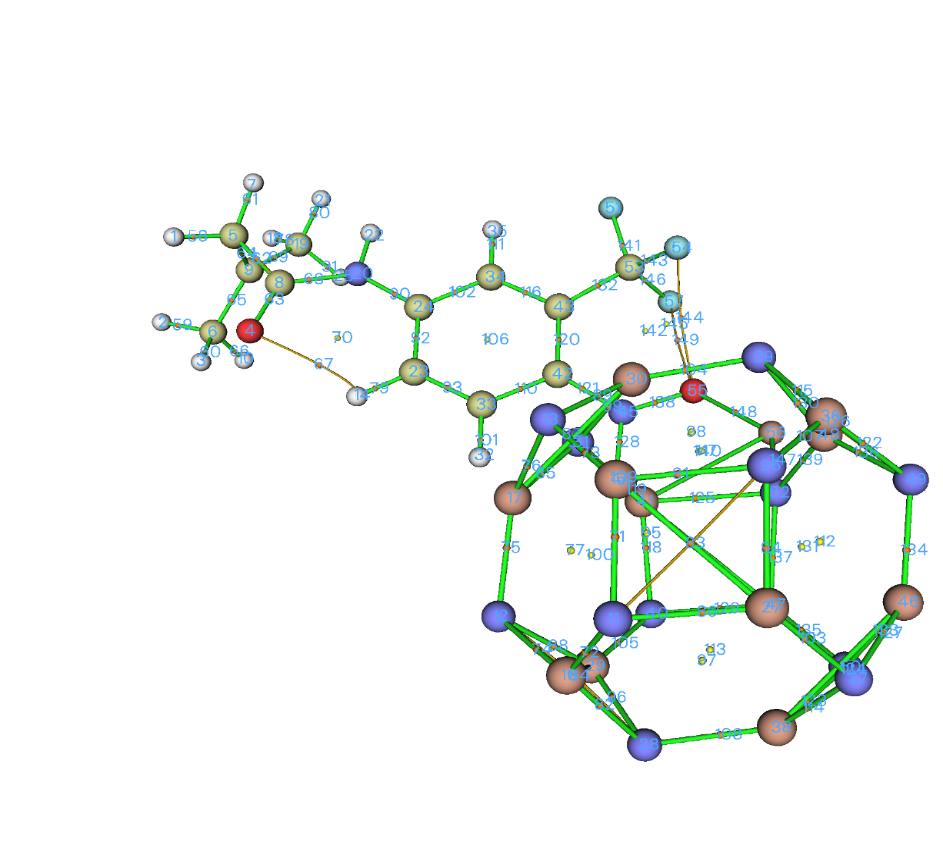

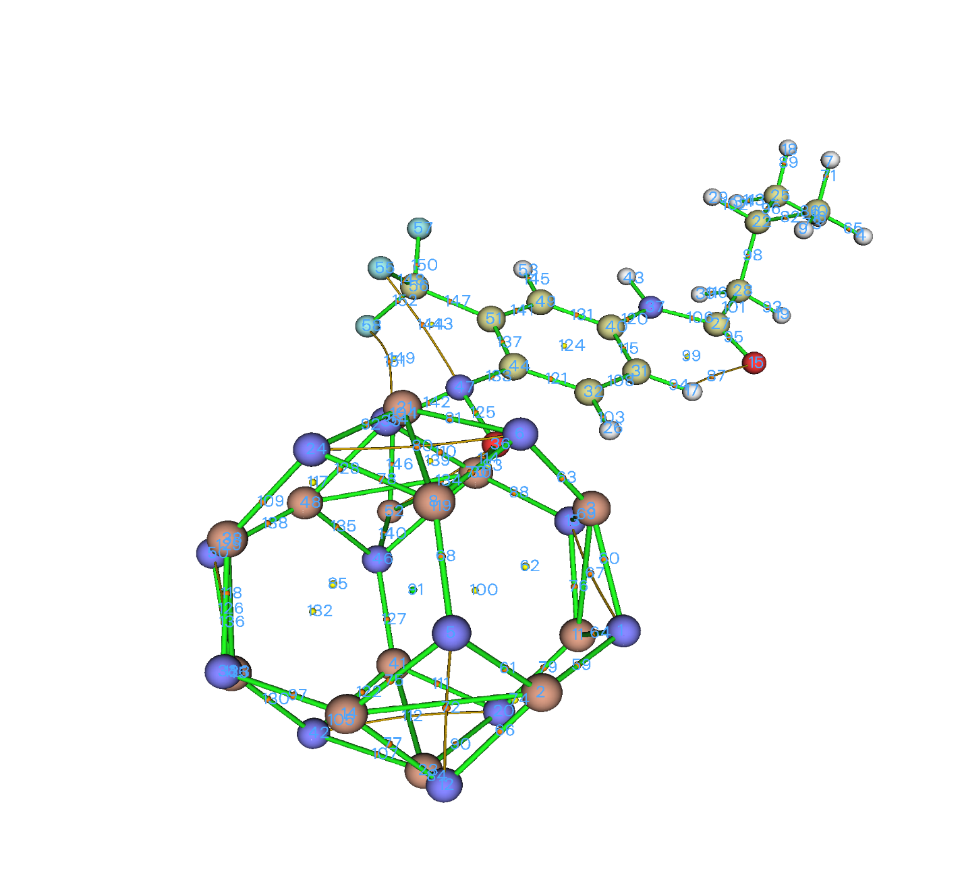


***Al_12_N_12_@Cu_FLU***

***Al_12_N_12_@Ni_FLU***

***Al_12_N_12_@Zn_FLU***

**FIGURE S1.** The QTAIM molecular graphs for all studied complexes. The bond critical points (BCPs) interactions represented with green lines while the brown lines indicate the interactions.
